# Impact of Risk Factors on Different Interval Cancer Subtypes in a Population-Based Breast Cancer Screening Programme

**DOI:** 10.1371/journal.pone.0110207

**Published:** 2014-10-21

**Authors:** Jordi Blanch, Maria Sala, Josefa Ibáñez, Laia Domingo, Belén Fernandez, Arantza Otegi, Teresa Barata, Raquel Zubizarreta, Joana Ferrer, Xavier Castells, Montserrat Rué, Dolores Salas, for the INCA Study Group

**Affiliations:** 1 Department of Epidemiology and Evaluation, IMIM (Hospital del Mar Medical Research Institute), Barcelona, Spain; 2 European Higher Education Area (EHEA) Doctoral Programme in Public Health in Department of Pediatrics, Obstetrics and Gynecology, Preventive Medicine and Public Health, Universitat Autònoma de Barcelona (UAB), Bellaterra, Barcelona, Spain; 3 Research network on health services in chronic diseases (REDISSEC), Barcelona, Spain; 4 General Directorate Public Health, Valencia, Spain; 5 Centre for Public Health Research (CSISP), FISABIO, Valencia, Spain; 6 Galician Breast Cancer Screening Programme, Directorate for innovation and management of public health, Santiago de Compostela, Spain; 7 Osakidetza Breast Cancer Screening Programme, Basque Country Health Service, Bilbao, Spain; 8 General Directorate of Health Care Programmes, Canary Islands Health Service, Las Palmas de Gran Canaria, Spain; 9 Department of Radiology, Hospital de Santa Caterina, Salt, Girona, Spain; 10 Department of Basic Medical Sciences, Biomedical Research Institut of Lleida (IRBLLEIDA)-University of Lleida, Lleida, Spain; University of North Carolina School of Medicine, United States of America

## Abstract

**Background:**

Interval cancers are primary breast cancers diagnosed in women after a negative screening test and before the next screening invitation. Our aim was to evaluate risk factors for interval cancer and their subtypes and to compare the risk factors identified with those associated with incident screen-detected cancers.

**Methods:**

We analyzed data from 645,764 women participating in the Spanish breast cancer screening program from 2000–2006 and followed-up until 2009. A total of 5,309 screen-detected and 1,653 interval cancers were diagnosed. Among the latter, 1,012 could be classified on the basis of findings in screening and diagnostic mammograms, consisting of 489 true interval cancers (48.2%), 235 false-negatives (23.2%), 172 minimal-signs (17.2%) and 114 occult tumors (11.3%). Information on the screening protocol and women's characteristics were obtained from the screening program registry. Cause-specific Cox regression models were used to estimate the hazard ratios (HR) of risks factors for interval cancer and incident screen-detected cancer. A multinomial regression model, using screen-detected tumors as a reference group, was used to assess the effect of breast density and other factors on the occurrence of interval cancer subtypes.

**Results:**

A previous false-positive was the main risk factor for interval cancer (HR = 2.71, 95%CI: 2.28–3.23); this risk was higher for false-negatives (HR = 8.79, 95%CI: 6.24–12.40) than for true interval cancer (HR = 2.26, 95%CI: 1.59–3.21). A family history of breast cancer was associated with true intervals (HR = 2.11, 95%CI: 1.60–2.78), previous benign biopsy with a false-negatives (HR = 1.83, 95%CI: 1.23–2.71). High breast density was mainly associated with occult tumors (RRR = 4.92, 95%CI: 2.58–9.38), followed by true intervals (RRR = 1.67, 95%CI: 1.18–2.36) and false-negatives (RRR = 1.58, 95%CI: 1.00–2.49).

**Conclusion:**

The role of women's characteristics differs among interval cancer subtypes. This information could be useful to improve effectiveness of breast cancer screening programmes and to better classify subgroups of women with different risks of developing cancer.

## Introduction

Evaluation of the impact of population-based breast cancer screening programmes is complex and can only be achieved in the long term. Regular screening contributes to reducing mortality from breast cancer, but also has adverse effects, such as false-positives, overdiagnosis and interval cancers [1], [2]. The European guidelines for quality assurance in breast cancer screening and diagnosis recommend the use of early performance indicators that provide information of the impact of screening [3]. These indicators should measure positive and negative effects to achieve a long-term balance between benefits and harms. One of the most important surrogate indicators of the effectiveness of breast cancer screening programmes is the interval cancer rate.

Interval cancers are primary breast cancers diagnosed in women after a negative screening test and before the next screening invitation [3]. Because they are diagnosed by symptoms, affected women lose the benefit of early detection and, in case of false-negative results, suffer delayed diagnosis and treatment.

Numerous studies have examined interval cancer rates in distinct screening programmes [4]–[13], allowing comparison of the sensitivity of these programmes. However, most studies do not distinguish among interval cancer subtypes, which can only be achieved after a radiological review of both screening and diagnostic mammograms. Half of interval cancers are true interval cancers in our context [14], which are tumours that are undetectable in the last screening participation but become symptomatic before the next participation. Failure to detect these tumours is caused by the limitations of the screening test and is inherent to organized screening process. Retrospectively, false-negative cancers (i.e., missed) can be seen in the screening mammogram and their occurrence is associated with the organization of screening programmes [14]–[16]. The remaining subtypes are minimal-sign cancers (which show detectable but non-specific sign at latest screen) and occult tumours at mammography (no signs of mammographic abnormalities either at screening or at diagnostic), which have been less studied since they account for less than 25% of interval cancers [14].

Study of interval cancers has focused on detecting differences with screen-detected cancers according to personal and tumor-related characteristics [14], [16]–[18]. Compared with screen-detected cancers, interval cancers show a higher prevalence of features associated with poor prognosis [14], [16]. Breast density has also been related to interval cancer [17], [19], showing a positive association between higher breast density and interval cancer risk. However, the specific role of breast density on interval cancer subtypes taking into consideration personal and organizational characteristics has not been evaluated.

The aim of our study was to evaluate the factors associated with a higher probability of interval cancer (overall, true interval, false negative, minimal signs and occult tumours) and to compare them with those associated with incident screen-detected cancers. We also studied the influence of breast density on detection mode and the occurrence of subtypes.

## Methods

### Ethics Statement

The study was approved by the Mar Teaching Hospital Research Ethics Committee. The data was analyzed anonymously and therefore no additional informed consent was required. Further information about our data and the methods used can be requested from the authors.

### Settings and study population

Population-based breast cancer screening in Spain is offered individually to 100% of the target population by the National Health Service. This programme adheres to the European Guidelines [3] and its results meet the required standards [20]. Each of the 17 administrative regions in Spain is responsible for the local application and has several radiology units that carry out screening. Despite the high degree of consensus, regional application can vary in the target population definition and in the mammographic screening protocol used (starting age, single or double reading, etc) [20].

All women aged 50 years (or 45 years depending on the region) to 69 years are actively invited to participate by letter every 2 years [20]. The screening programmes stop inviting women with a breast cancer diagnosis.

**Figure 1:**
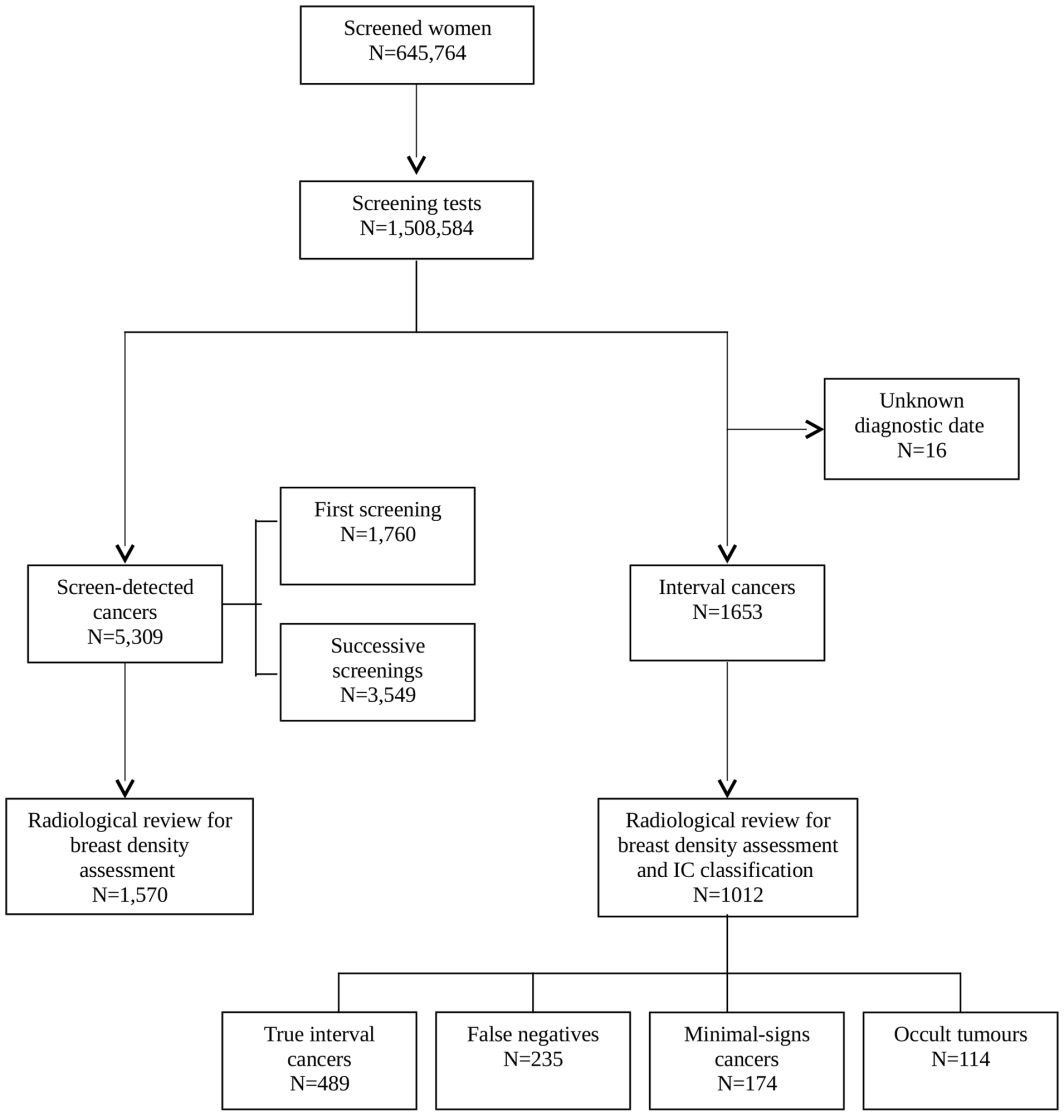
Flow chart of the study populations.

We built a retrospective cohort of 645,764 screened women from 32 radiology units in five regions of Spain who underwent mammography between January 1, 2000 and December 31, 2006 and who were followed up until June 30, 2009 for interval cancer identification. These women underwent a total of 1,508,584 screening mammograms (Figure 1). During the study period, 5,309 cancers were detected in routine screening mammograms, of which 3,547 were detected in successive participations, and 1,653 emerged as interval cancers. Interval cancers with unknown diagnostic date (n = 16) were excluded. We included both invasive and in situ carcinomas.

### Screening outcomes

Screening mammography has three possible outcomes: a negative result, a positive result (abnormal findings requiring further assessments) or early recall (an intermediate mammogram is performed out of sequence with the screening interval at 6 or 12 months). Cancers detected at intermediate mammogram were considered screen-detected cancers [3].

A positive result is considered to be screen-detected tumour if, after further assessments, there is histopathological confirmation of cancer. Otherwise, the result is considered a false-positive and woman is invited at 2 years. Further assessments can include non-invasive procedures (magnetic resonance imaging, ultrasonography, additional mammography) and/or invasive (fine-needle aspiration cytology, core-needle biopsy and open biopsy).

Interval cancer was defined as “a primary breast cancer arising after a negative screening episode and before the next invitation to screening or within 24 months for women who reached the upper age limit” [3]. We extended the definition until the 30th month, because we allowed a 6 month margin for women to attend each round. Interval cancers were identified by merging data from the registers of screening programmes with population-based cancer registries [21], the regional Minimum Data Set (based on hospital discharges with information on the main diagnosis) and hospital-based cancer registries.

### Interval cancer classification and breast density assessment

Interval cancers were classified by three panels, formed by three experienced radiologists. The panels performed a semi-informed independent review of both screening and diagnostic mammograms with independent double reading and arbitration. Of 1,653 interval cancers, 1,012 had available screening and diagnostic mammograms, and therefore could be classified into the four interval cancer categories. Briefly, screening mammograms were first reviewed alone and provisionally classified into positive, negative, and minimal-signs. Afterwards, the screening and diagnostic mammograms were reviewed together and interval cancers were definitively classified into: true interval cancers (the screening mammogram showed normal or benign results), false-negatives (an abnormality suspicious for malignancy was retrospectively seen on the screening mammogram), minimal-signs (detectable but non-specific signs were identified on the screening mammogram) and occult tumours (showing no mammographic abnormalities at diagnosis despite clinical signs) [3]. More details of the classification process have been reported in a previous study [15].

The breast density of the cancer-free breast was determined by one radiologist from each panel, using Boyd's scale, a semi-quantitative score of six categories using percentages of density: A: 0%; B: 1–10%; C:10–25%; D:25–50%; E:50–75%; F:75–100% [22]. For purposes of this study, the first three categories were combined into the <25% group [23]. Breast density was assessed for classified interval cancers (N = 1,012) and from a sample of screen-detected cancers matched by region and year of the last screening (N = 1,570).

### Study variables

All information was collected from each woman at each attendance. The variables related to the screening protocol included the reading method (single or double reading) and mammography type (screen-film mammography [SFM] or digital mammography [DM]). The variables related to women's personal characteristics were use of hormonal replacement therapy (HRT) at screening or in the previous 6 months (Yes/No), menopausal status (pre- or postmenopausal), previous benign biopsy outside screening (Yes/No), the presence or absence of a first-degree familial history of breast cancer (Yes/No), mammography date, number of participations, the existence of a previous false-positive (Yes/No), and the existence of an early recall (Yes/No).

### Statistical analysis

Breast cancer rates per 1,000 women and per 10,000 mammograms were estimated, for interval cancers and for screen-detected cancers.

The cumulative probability of suffering an interval cancer was calculated using the Kaplan-Meier estimator with time-dependent variables according the distinct study variables. We estimated the hazard ratios (HR) of interval cancer and incident screen-detected cancer using a multivariate cause-specific Cox model with time-dependent variables. Radiology units were included as a random effect [24]. We built explanatory models including all relevant covariates according to the literature to evaluate their effect and statistical significance on the risk of cancer detection and of interval cancer subtypes.

Given that incidence of breast cancer depends on age, we used age as time scale in the Kaplan-Meier and Cox regression analyses [25]. Age at study entry was defined as the age at the first mammogram in the study period. Age at study exit was the lowest of: age at breast cancer diagnosis, age at the study closure, or age at 30 months after the last mammogram. Observations were censored when the event breast cancer diagnosis did not occur during the study period.

We conducted a case-control analysis to determine whether the study variables differed between interval cancers (cases) and screen-detected cancers (controls), using a multivariate logistic regression model. For the analysis of variables associated to the subtypes of interval cancer, we fitted a multinomial regression model, using the screen-detected cancers as a reference group. The multinomial model estimates k-1 models, comparing each group to the referent group. The exponentiated multinomial logit coefficient provides an estimate of the relative risk and, commonly, is expressed as relative risk ratio (RRR).

All P-values were based on two-sided tests and were considered statistically significant if less than 0.05. Statistical analyses were performed using the R statistical software (version 3.0.1) [26].

## Results

### Interval cancer subtypes and risk factors

The interval cancer rate was 2.57 per 1,000 screened women and 10.99 per 10,000 screening mammograms (Table 1). The largest proportion of interval cancers was diagnosed in the second year (60.0% of interval cancers). The radiologist teams classified 1,012 interval cancers as follows: 489 true intervals, 235 false-negatives, 174 minimal-signs and 114 occult tumours.

**Table 1:** Number of screen-detected and interval cancers (by time of diagnosis) with their rates per 1,000 women and 10,000 mammograms and proportions of interval cancer subtypes among radiologically classified interval cancers.

		Rates per 1,000 women	Rates per 10,000 Mammograms	Radiologic information	TI	FN	MS	OT
	*N*			*N*	*N*	*N*	*N*	*N*
**Screened women**	645,764							
**Mammograms**	1,508,584							
**Screen-detected cancer**	5,307	8.22	35.18	1,570				
In successive participations	3,547	5.49	23.54	1,100				
**Interval cancer**	1,653	2.57	10.99	1,012	489	235	174	114
Time of diagnosis*	N (%)			N (%)	N (%)	N (%)	N (%)	N (%)
<12 months	484 (29.3)	0.75	3.21	273 (27.0)	96 (19.6)	76 (32.3)	53 (30.5)	48 (42.1)
12–23 months	992 (60.0)	1.54	6.58	626 (61.9)	322 (65.8)	139 (59.1)	105 (60.3)	60 (52.6)
> = 24 months	177 (10.7)	0.27	1.17	113 (11.2)	71 (14.5)	20 (8.5)	16 (9.2)	6 (5.3)

*Time of diagnosis (in months) for interval cancer is the time between the last mammogram and diagnosis of cancer.

**Abbreviations**: TI: True interval, FN: False-negative, MS: Minimal-signs, OT: Occult tumours.

Among classified interval cancers, 42.1% of occult tumours, 32.3% of false-negatives and 31.0% of minimal-signs were diagnosed in the first 12 months after the participation and only 19.6% of true interval cancers emerged in the same period.

**Figure 2:**
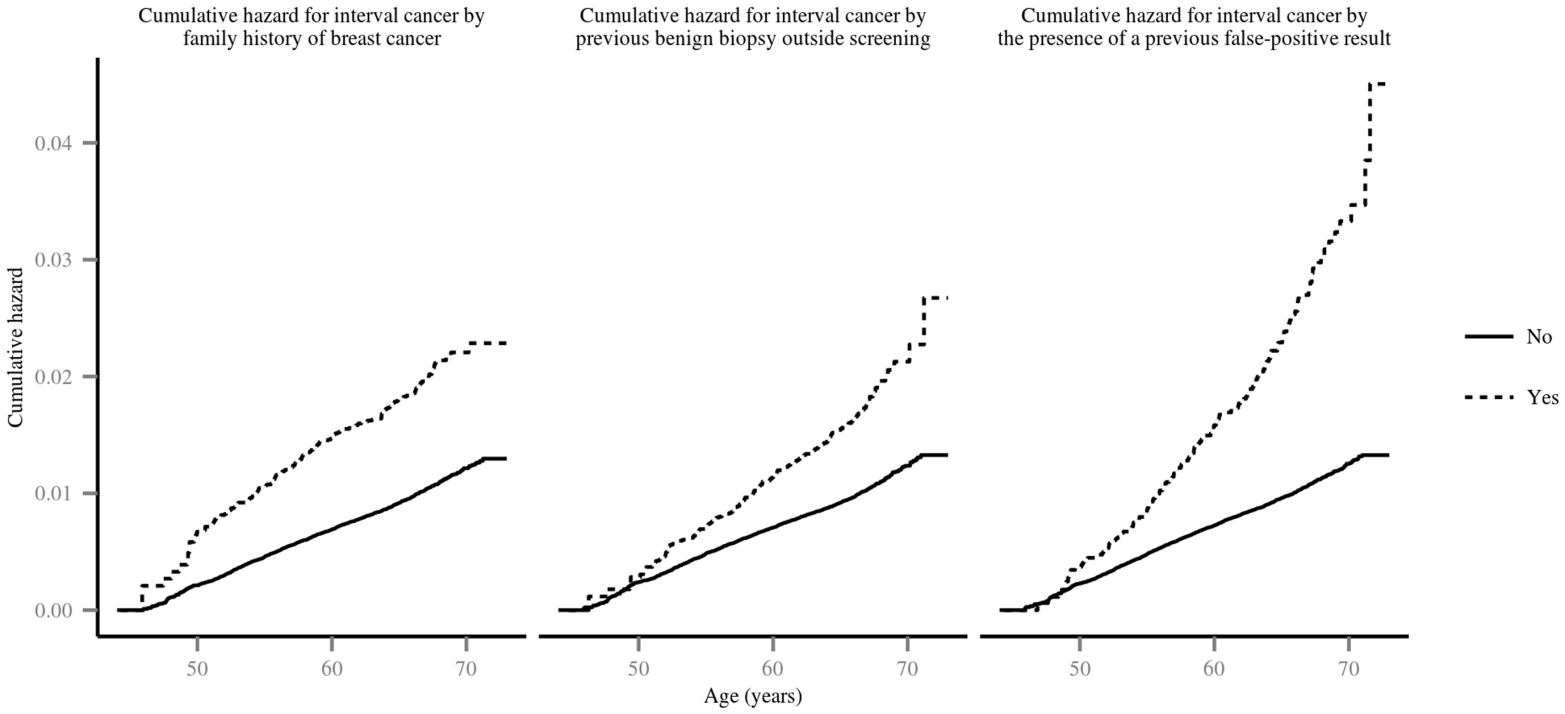
Cumulative hazard for interval cancer by the presence of a previous false-positive result, family history of breast cancer and previous benign biopsy outside screening.

Figure 2 presents the cumulative probability of developing an interval cancer according to family history of breast cancer, previous benign biopsy outside screening and the presence of a previous false-positive result. The other covariates did not show statistically significant differences in the bivariate analysis (data not shown). Women with a false-positive result in the previous participation had a higher cumulative probability of developing interval cancer than women without (0.033 vs 0.013). Women with family history of breast cancer or previous benign biopsy result outside screening had a higher cumulative probability than women without (0.021 vs 0.012 and 0.022 vs 0.012, respectively).

The hazard risks from cause-specific survival analyses for incident screen-detected cancers and interval cancers (overall and subtypes) are shown in Table 2 (see also tables S1 and S2). The existence of a false-positive in the previous mammogram showed the highest hazard ratio for developing interval cancer (HR = 2.71; 95% CI: 2.28–3.23), while having an early recall showed the highest hazard ratio for screen-detected cancer (HR = 3.59; 95% CI:3.12–4.14). A previous false-positive result, premenopausal status, a family history of breast cancer and previous benign breast biopsy were risk factors for both interval and screen-detected cancers. HRT use was significantly associated with increased risk of developing interval cancer (HR = 1.27; 95% CI:1.07–1.5). DM was associated with screen-detected cancer (HR = 1.34; 95% CI:1.12–1.61). Early recall was a protective factor for interval cancer (HR = 0.56; 95% CI:0.40–0.78).

**Table 2:** Adjusted hazard ratios from cause-specific survival analyses for incident screen-detected cancers and for interval cancer (overall and subtypes).

	Incident SDC	IC	TI	FN	MS	OT
	HR* (95%CI)	HR* (95%CI)	HR* (95%CI)	HR* (95%CI)	HR* (95%CI)	HR* (95%CI)
**Reading Method**						
Double	Ref	Ref	Ref	Ref	Ref	Ref
Single	1.31 (0.85–2.02)	0.85 (0.60–1.21)	1.55 (0.66–3.62)	1.09 (0.36–3.25)	1.38 (0.26–7.28)	0.52 (0.15–1.81)
**Type of mammogram**						
SFM	Ref	Ref	Ref	Ref	Ref	Ref
DM	1.34 (1.12–1.61)	1.00 (0.78–1.28)	2.17 (1.37–3.45)	1.57 (0.78–3.13)	0.97 (0.35–2.67)	0.36 (0.11–1.24)
**Early Recall**						
No	Ref	Ref	Ref	Ref	Ref	+
Yes	3.59 (3.12–4.14)	0.56 (0.40–0.78)	0.46 (0.22–0.96)	0.36 (0.15-0.83)	0.85 (0.29–2.46)	
**Previous false-positive**						
No	Ref	Ref	Ref	Ref	Ref	Ref
Yes	1.34 (1.20–1.60)	2.71 (2.28–3.23)	2.26 (1.59–3.21)	8.79 (6.24–12.40)	1.80 (0.93–3.47)	0.34 (0.08–1.39)
**HRT use**						
No	Ref	Ref	Ref	Ref	Ref	Ref
Yes	0.98 (0.86–1.11)	1.27 (1.07–1.50)	1.33 (0.99–1.79)	1.30 (0.82–2.05)	1.63 (1.03–2.56)	0.92 (0.46–1.84)
**Menopausal status**						
Postmenopausal	Ref	Ref	Ref	Ref	Ref	Ref
Premenopausal	1.26 (1.09–1.45)	1.41 (1.20–1.67)	1.29 (0.94–1.76)	1.28 (0.79–2.07)	0.91 (0.52–1.61)	1.33 (0.78–2.27)
**Family history of breast cancer**						
No	Ref	Ref	Ref	Ref	Ref	Ref
Yes	1.75 (1.56–1.94)	1.65 (1.41–1.93)	2.11 (1.60–2.78)	1.44 (0.91–2.27)	1.67 (1.02–2.74)	1.44 (0.77–2.69)
**Previous benign biopsy outside screening**						
No	Ref	Ref	Ref	Ref	Ref	Ref
Yes	1.34 (1.18–1.53)	1.73 (1.46–2.04)	1.26 (0.90–1.74)	1.83 (1.23–2.71)	1.44 (0.87–2.37)	1.12 (0.54–2.33)

*Multivariate Cox time-dependent models. The hazard ratios for each variable were adjusted for the other variables in the table, the year of the last mammogram, and for radiology unit (as a random effect).

+We excluded early recall, because there were no cases of early recall in occult tumors.

**Abbreviations**: SDC: Screen-detected cancer, IC: Interval Cancer, TI: True interval, FN: False-negative, MS: Minimal-signs, OT: Occult tumors, SFM: Screen-film mammography, DM: Digital mammography and HRT: Hormonal Replacement Therapy.

The effect of the risk factors on developing interval cancer differed according to subtypes. DM was associated with true interval cancer (HR = 2.17; 95% CI:1.37–3.45), but not with false-negatives (HR = 1.57; 95% CI:0.78–3.13). The effect of a previous false-positive result was higher for false-negatives (HR = 8.79; 95% CI:6.24–12.40) than for true interval cancer (HR = 2.26; 95% CI:1.59–3.21). HRT use was a statistically significant risk factor for minimal-signs (HR = 1.63; 95% CI:1.03–2.56) and a family history of breast cancer was a risk factor for true interval cancer (HR = 2.11; 95% CI:1.60–2.78) and minimal-signs as well (HR = 1.67; 95% CI:1.02–2.74). Previous benign biopsy outside screening was a risk factor for a false-negative cancers (HR = 1.83; 95% CI:1.23–2.71).

### Comparison of breast density and other risk factors between interval cancers and screen detected cancers

The proportion of women with extremely dense breasts (Table 3) was higher for interval cancers than for screen-detected cancers (16.4% and 11.7%, respectively, P<0.001); the highest proportion was found in occult tumours (28.1%) and the lowest in minimal-signs cancers (9.8%).

**Table 3:** Distribution of variables related to the screening protocol and women's characteristics by screen-detected and interval cancer (overall and subtypes).

	SDC	IC	TI	FN	MS	OT
	*N* (%)	*N* (%)	*N* (%)	*N* (%)	*N* (%)	*N* (%)
**Total**	1,570	1,012	489	235	174	114
**Reading method**						
Double	1,450 (92.4)	889 (87.8)*	424 (86.7)	202 (86.0)	162 (93.1)	101 (88.6)+
Single	120 (7.6)	123 (12.2)	65 (13.3)	33 (14.0)	12 (6.9)	13 (11.4)
**Type of Mammogram**						
SFM	1,470 (93.6)	941 (93.0)	446 (91.2)	216 (91.9)	168 (96.6)	111 (97.4)+
DM	100 (6.4)	71 (7.0)	43 (8.8)	19 (8.1)	6 (3.4)	3 (2.6)
**Participation**						
Successive	1,100 (70.1)	742 (73.3)	361 (73.8)	176 (74.9)	128 (73.6)	77 (67.5)
Initial	470 (29.9)	270 (26.7)	128 (26.2)	59 (25.1)	46 (26.4)	37 (32.5)
**Early Recall**						
No	1,423 (90.6)	985 (97.3)*	474 (96.9)	227 (96.6)	170 (97.7)	114 (100.0)+
Yes	147 (9.4)	27 (2.7)	15 (3.1)	8(3.4)	4 (2.3)	0 (0.0)
**Previous false-positive**						
No	1,475 (93.9)	904 (89.3)*	444 (90.8)	185 (78.7)	163 (93.7)	112 (98.2)+
Yes	95 (6.1)	108 (10.7)	45 (9.2)	50 (21.3)	11 (6.3)	2 (1.8)
**Age group (yrs)**						
44–49	81 (5.2)	61 (6.1)*	32 (6.5)	12 (5.1)	8 (4.6)	10 (8.8)+
50–54	421 (26.8)	351 (34.7)	170 (34.8)	73 (31.1)	54 (31.0)	54 (47.4)
55–59	438 (27.9)	271 (26.8)	136 (27.8)	57 (24.3)	55 (31.6)	23 (20.2)
60–64	460 (29.3)	224 (22.1)	103 (21.1)	62 (26.4)	39 (22.4)	20 (17.5)
65–70	170 (10.8)	104 (10.3)	48 (9.8)	31 (13.2)	18 (10.3)	7 (6.1)
**HRT use**						
No	1,306 (91.5)	771 (87.9)*	366 (87.6)	175 (88.8)	139 (85.8)	91 (91.0)+
Yes	122 (8.5)	106 (12.1)	52 (12.4)	22 (11.2)	23 (14.2)	9 (9.0)
**Menopausal status**						
Postmenopausal	1,222 (85.5)	725 (81.7)*	346 (81.6)	166 (83.0)	138 (86.8)	75 (72.1)+
Premenopausal	207 (14.5)	162 (18.3)	78 (18.4)	34 (17.0)	21 (13.2)	29 (27.9)
**Family history of breast cancer**						
No	1,224 (87.5)	760 (87.5)	352 (85.6)	175 (89.3)	139 (88.5)	94 (89.5)
Yes	175 (12.5)	109 (12.5)	59 (14.4)	21 (10.7)	18 (11.5)	11 (10.5)
**Previous benign biopsy outside screening**						
No	1,168 (88.1)	717 (88.0)	347 (89.4)	158 (83.6)	134 (88.1)	78 (90.7)
Yes	158 (11.9)	98 (12,0)	41 (10.6)	31 (16.4)	18 (11.8)	8 (9.3)
**Density**						
<25%	607 (38.7)	325 (32.1)*	153 (31.3)	86 (36.6)	68 (39.1)	18 (15.8)+
25–50%	428 (27.3)	169 (26.6)	134 (27.4)	64 (27.2)	49 (28.2)	22 (19.3)
50–75%	352 (22.4)	252 (24.9)	123 (25.2)	47 (20.0)	40 (23.0)	42 (36.8)
>75%	183 (11.7)	166 (16.4)	79 (16.2)	38 (16.2)	17 (9.8)	32 (28.1)

*P-value<0.05, Chi-squared test between screen-detected and interval cancer.

+*P*-value<0.05, Chi-squared test between screen-detected and interval cancer subtype.

**Abbreviations**: SDC: Screen-detected Cancer, IC: Interval Cancer, TI: True interval, FN: False-negative, MS: Minimal-signs, OT: Occult tumours, SFM: Screen-film mammography, DM: Digital mammography and HRT: Hormone Replacement Therapy.

Table 4 presents the risk factors associated with interval cancers compared with screen-detected cancers. Risk factors for overall interval cancer were the presence of a previous false-positive (OR = 2.11; 95% CI:1.56–2.86), HRT use (OR = 1.57; 95% CI:1.18–2.10) and extremely dense breasts (OR = 1.63; 95% CI:1.24–2.14). By interval cancer subtypes, risk factors for true interval cancer were the same as those for interval cancer: the presence of a previous false-positive (RRR = 1.79; 95% CI:1.21–2.63), HRT use (RRR = 1.57; 95% CI:1.10–2.25) and extremely dense breasts (RRR = 1.67; 95% CI:1.18–2.36). However, for false negatives, only the presence of a previous false-positive result was a risk factor (RRR = 4.55; 95% CI:3.07–6.75), which was the strongest observed association in this analysis. Extremely breast density was at the limit of significance (RRR = 1.58; 95% CI:1.00–2.49). The only statistically significant risk factor for minimal signs was HRT use (RRR = 1.84; 95% CI:1.12–3.02), and that for occult tumours was extremely breast density (RRR = 4.92; 95% CI:2.58–9.38).

**Table 4:** Adjusted odds ratio (OR) or relative risk ratios (RRR) for interval cancer and subtypes compared with screen-detected cancers.

	IC	TI	FN	MS	OT
	OR* (95%CI)	RRR** (95%CI)	RRR** (95%CI)	RRR** (95%CI)	RRR** (95%CI)
**Reading method**					
Single vs Double	1.55 (0.84–2.91)	1.60 (0.75–3.42)	1.67 (0.62–4.55)	2.09 (0.45–9.75)	1.03 (0.24–4.47)
**Type of Mammogram**					
DM vs SFM	0.61 (0.40–0.90)	0.84 (0.52–1.37)	0.56 (0.29–1.11)	0.37 (0.14–0.99)	0.21 (0.06–0.79)
**Participation**					
Initial vs Successive	0.70 (0.57–0.87)	0.66 (0.50–0.87)	0.78 (0.53–1.13)	0.77 (0.51–1.16)	0.65 (0.40–1.06)
**Early Recall**					
Yes vs No	0.23 (0.14–0.35)	0.27 (0.15–0.47)	0.25 (0.11–0.55)	0.23 (0.08–0.64)	+
**Previous false-positive**					
Yes vs No	2.11 (1.56–2.86)	1.79 (1.21–2.63)	4.55 (3.07–6.75)	1.15 (0.59–2.21)	0.32 (0.08–1.33)
**Age group (yrs)**					
44–49 vs 50–54	1.07 (0.71–1.59)	1.17 (0.71–1.91)	0.87 (0.42–1.79)	1.05 (0.45–2.43)	1.17 (0.53–2.59)
55–59 vs 50–54	0.65 (0.51–0.82)	0.65 (0.48–0.88)	0.69 (0.45–1.06)	0.80 (0.51–1.26)	0.37 (0.21–0.66)
60–64 vs 50–54	0.52 (0.40–0.67)	0.49 (0.35–0.67)	0.71 (0.46–1.10)	0.53 (0.32–0.87)	0.36 (0.19–0.66)
65–70 vs 50–54	0.65 (0.47–0.89)	0.61 (0.41–0.92)	0.92 (0.54–1.55)	0.67 (0.36–1.24)	0.31 (0.13–0.75)
**HRT use**					
Yes vs No	1.57 (1.18–2.10)	1.57 (1.10–2.25)	1.48 (0.89–2.44)	1.84 (1.12–3.02)	1.26 (0.60–2.64)
**Menopausal status**					
Pre vs Postmenopausal	0.91 (0.67–1.22)	0.91 (0.63–1.32)	1.00 (0.60–1.68)	0.77 (0.42–1.40)	0.84 (0.46–1.53)
**Family history of breast cancer**					
Yes vs No	0.98 (0.75–1.28)	1.16 (0.84–1.61)	0.80 (0.49–1.31)	0.91 (0.54–1.53)	0.75 (0.38–1.46)
**Previous benign biopsy outside screening**					
Yes vs No	0.89 (0.67–1.17)	0.77 (0.53–1.12)	1.19 (0.77–1.85)	0.94 (0.55–1.60)	0.70 (0.32–1.49)
**Density**					
25–50% vs <25%	1.18 (0.95–1.46)	1.26 (0.96–1.66)	1.12 (0.78–1.61)	0.99 (0.66–1.47)	1.53 (0.80–2.94)
50–75% vs <25%	1.28 (1.03–1.60)	1.33 (1.00–1.77)	0.97 (0.65–1.44)	0.93 (0.61–1.43)	3.54 (1.97–6.36)
>75% vs <25%	1.63 (1.24–2.14)	1.67 (1.18–2.36)	1.58 (1.00–2.49)	0.74 (0.41–1.34)	4.92 (2.58–9.38)

The last category in each row is the reference category.

*Logistic regression between screen-detected (ref) and interval cancer. The ORs are adjusted for the other variables in the table and the year of last mammogram.

**Multinomial regression between screen-detected (ref) and subtypes of interval cancer. The RRRs are adjusted for the other variables in the table and the year of last mammogram.

+We excluded early recall, because there were no cases of early recall in occult tumors. All the estimates are also adjusted by mammogram year.

**Abbreviations**: SDC: Screen-detected cancer, IC: Interval Cancer, TI: True interval, FN: False-negative, MS: Minimal-signs, OT: Occult tumours, SFM: Screen-film mammography, DM: Digital mammography and HRT: Hormone Replacement Therapy.

## Discussion

This study provides information on the determinants of interval cancer and its subtypes and a comparison with screen-detected cancer in a retrospective cohort of screened women. Women's characteristics (premenopausal status, a family history of breast cancer, and previous benign breast biopsy) were risk factors for both interval and screen-detected cancer, showing a similar strength of association. A family history of breast cancer had the strongest association with true interval cancer while previous benign biopsy was a significant risk factor only for false-negatives. The presence of a previous false-positive result was a risk factor for both screen-detected and interval cancer but the association with interval cancer was stronger, especially for false-negative cancers. Double reading, digital mammography and early recall were associated with screen-detected cancer but not with interval cancer.

Few studies have analysed the determinants of interval cancer and even fewer have taken subtypes into account. Previous studies have described interval cancer rates ranging from 1.8 to 2.9 per 1,000 women and from 10.6 to 29.5 per 10,000 mammograms [4]–[13], which are consistent with our results. The main risk factor for interval cancer was a previous false-positive result, with the strongest association for false-negatives, suggesting that some results interpreted as false-positives may, in fact, be false-negatives [27]–[29]. In fact, false positive have been found also a risk factor for screen cancer detection especially when cytology or biopsy had been performed as further assessment [30], [31].

DM was not associated with increased risk of interval cancer, in line with the study by Hoff et al. [32] and a recent study from Norway [33], showing that DM use did not modify false-negatives rates. The increased risk of true interval cancers with DM could be explained by the higher precision in detecting less advanced and smaller tumours with DM, which has been demonstrated in several studies carried out in mammography screening contexts [32]–[35].

Premenopausal status, a family history of breast cancer and previous benign breast biopsy result are well-known risk factors for breast cancer [36], which is coherent with our findings and those of previous studies, revealing an association with both incident screen-detected and interval cancer [9], [18], [37]. However, the role of these factors seemed to differ when we specifically analysed subtypes. HRT use was a risk factor for minimal-signs, but we have no information on HRT type or on treatment length, which could have affected these findings [37]. The effect of family history was greater for true interval cancer, in agreement with the hypothesis that tumours in women with a family history of breast cancer grow faster and are more aggressive [16], [38].

Because information on breast density was not available for the whole cohort, we analyzed the effect of breast density as a risk factor for interval cancer subtypes compared with those of screen-detected cancers in a case-control. In previous study in the same cohort we explored the role of breast density within interval cancer subtypes, adjusting by tumour characteristics [15]. In the current work, we considered women-related and organizational characteristic, obtaining consistent results. High breast density was a risk factor for interval cancer, mainly for occult tumours, but also for true interval and false-negatives. Pollan et al. observed that breast density played a greater role in interval cancer than in screen-detected cancer [19]. The strong association of breast density and occult tumours pointed to a masking effect, but breast density appears to play a lesser role in false-negatives. The association with true interval cancer also reinforce the hypothesis that tumours stimulated by growth factors found in dense breasts [39] are more likely to be true interval cancers. Understanding the role of breast density is important in breast cancer screening since it is one of the variables proposed to tailor screening.

This study has some limitations. First, we could not get all diagnostic mammograms from interval cancers mainly because logistic reasons, avoiding a complete interval cancer classification. Moreover, misclassification among interval cancers cannot be excluded because is inherent to radiologist subjectivity. However, this misclassification would attenuate differences among study groups. Breast density was not available for the entire population because it was not systematically collected and registered. Therefore, we could not include this factor in the analysis of determinants. In addition, breast density was visually assessed, implying also some subjectivity and misclassification. Equally, information was unavailable on other important cancer-related variables such as age at menarche, age at maternity, the number of children and body mass index, type of mammographic abnormality and clinico-histopathological features of the breast cancers. The small numbers of occult tumours did not provide sufficient statistical power for this subgroup in some analyses.

A strength of study was the cohort size and the consolidation of the screening programmes in Spain. The participating radiology units had completed at least five rounds ensuring the quality indicators were stable. There are other studies with radiologically classified interval cancers, but the present is, to our knowledge, the largest one with information on breast density for the different subtypes.

In conclusion, the current work provides comprehensive data on the relationship between personal and organizational characteristics and the risk of interval cancer. This information could be useful to better classify subgroups of women at different risks of developing cancer in a moment when personalization of breast cancer screening is being proposed [40]. The strong relationship observed between false-positives results and false negatives, together with the previous knowledge on the relationship between false-positive and cancer, emphasizes the need for return for further screening in women with false-positive results, and calls for more research on this topic.

## Supporting Information

Table S1 **Incidence of variables related to the screening protocol and women's characteristics by screen-detected and interval cancer (overall and subtypes).**
(DOC)

Table S2 **Crude hazard ratios from cause-specific survival analyses for incident screen-detected cancers and for interval cancer (overall and subtypes).**
(DOC)
