# Supplementary material for: Impact of Risk Factors on Different Interval Cancer Subtypes in a Population-Based Breast Cancer Screening Programme
Source: PLoS One. 2014 Oct 21;9(10):e110207. doi: 10.1371/journal.pone.0110207 (PMC4204862; doi:10.1371/journal.pone.0110207)
Supplement: Table S1 — Incidence of variables related to the screening protocol and women's characteristics by screen-detected and interval cancer (overall and subtypes). (DOC) [file pone.0110207.s001.doc]

**Table S1.** Incidence of variables related to the screening protocol and women’s characteristics by screen-detected and interval cancer (overall and subtypes).

|  | **Total** | **Incident SDC** | **IC** | | **TI** | **FN** | | **MS** | **OT** | |
| --- | --- | --- | --- | --- | --- | --- | --- | --- | --- | --- |
|  | person-yrs | N (IR) | N (IR) | | N (IR) | N (IR) | | N (IR) | N (IR) | |
|  | 3,087,261 | 3,547 (1.15) | 1,653 (0.53) | | 489 (0.16) | 235 (0.08) | | 174 (0.06) | 114 (0.04) | |
| **Reading Method** |  |  |  | |  |  | |  |  | |
| Double | 2,581,430.6 | 2,859 (1.11) | 1,379 (0.53) | | 424 (0.16) | 202 (0.08) | | 162 (0.06) | 101 (0.04) | |
| Single | 505,830.2 | 688 (1.36) | 274 (0.54) | | 65 (0.13) | 33 (0.06) | | 13 (0.02) | 12 (0.02) | |
| **Type of mammogram** |  |  |  | |  |  | |  |  | |
| SFM | 2,931,388.4 | 3,290 (1.12) | 1,545 (0.53) | | 446 (0.15) | 216 (0.07) | | 168 (0.06) | 111 (0.04) | |
| DM | 155,872.4 | 257 (1.65) | 108 (0.69) | | 43 (0.27) | 19 (0.12) | | 6 (0.04) | 3 (0.02) | |
| **Early Recall** |  |  |  | |  |  | |  |  | |
| No | 3,001,825.4 | 3,263 (1.09) | 1,603 (0.53) | | 474 (0.16) | 227 (0.07) | | 170 (0.06) | 114 (0.04) | |
| Yes | 85,435.4 | 284 (3.32) | 50 (0.58) | | 15 (0.17) | 8 (0.09) | | 4 (0.05) | 0 (0.00) | |
| **Previous false-positive** |  |  |  | |  |  | |  |  | |
| No | 2,957,003.1 | 3,114 (1.05) | 1,483 (0.50) | | 444 (0.15) | 185 (0.06) | | 163 (0.05) | 112 (0.04) | |
| Yes | 130,245.1 | 282 (1.30) | 170 (1.30) | | 45 (0.34) | 50 (0.38) | | 11 (0.08) | 2 (0.01) | |
| Unknown | 12.6 | 151 (12,000) | 0 (0.00) | | 0 (0.00) | 0 (0.00) | | 0 (0.00) | 0 (0.00) | |
| **HRT use** |  |  |  | |  |  | |  |  | |
| No | 2,304,781.0 | 2,579 (1.12) | 1,195 (0.52) | | 366 (0.16) | 175 (0.08) | | 139 (0.06) | 91 (0.04) | |
| Yes | 268,426.0 | 272 (1.01) | 162 (0.60) | | 52 (0.19) | 22 (0.08) | | 23 (0.08) | 9 (0.03) | |
| Unknown | 514,053.8 | 696 (1.35) | 296 (0.58) | | 71 (0.14) | 38 (0.07) | | 12 (0.02) | 14 (0.03) | |
| **Menopausal status** |  |  |  | |  |  | |  |  | |
| Postmenopausal | 2,365,917.9 | 2,930 (1.24) | 1,170 (0.49) | | 346 (0.15) | 166 (0.07) | | 138 (0.06) | 75 (0.03) | |
| Premenopausal | 449,901.9 | 279 (0.62) | 284 (0.63) | | 78 (0.17) | 34 (0.7) | | 21 (0.05) | 29 (0.06) | |
| Unknown | 271,441.1 | 338 (1.24) | 199 (0.73) | | 65 (0.24) | 35 (0.13) | | 15 (0.05) | 10 (0.04) | |
| **Family history of breast cancer** |  |  |  |  | | |  |  | |  |
| No | 2,581,095.6 | 2,724 (1.05) | 1,256 (0.49) | | 352 (0.14) | 175 (0.07) | | 139 (0.05) | 94 (0.04) | |
| Yes | 219,548.5 | 430 (1.96) | 178 (0.81) | | 59 (0.27) | 21 (0.09) | | 18 (0.08) | 11 (0.05) | |
| Unknown | 286,616.7 | 393 (1.37) | 219 (0.76) | | 78 (0.27) | 39 (0.14) | | 17 (0.06) | 9 (0.03) | |
| **Previous benign biopsy outside screening** |  |  |  |  | | |  |  | |  |
| No | 2,105,848.0 | 2,143 (1.02) | 1,013 (0.48) | | 347 (0.16) | 158 (0.07) | | 134 (0.06) | 78 (0.04) | |
| Yes | 189,765.4 | 268 (1.41) | 166 (0.87) | | 41 (0.22) | 31 (0.16) | | 18 (0.09) | 8 (0.04) | |
| Unknown | 791,647.4 | 1,136 (1.43) | 474 (0.60) | | 101 (0.13) | 46 (0.06) | | 22 (0.03) | 28 (0.03) | |

Incidence rate was measured per 1,000 person-years.

**Abbreviations**: IR: Incidence rate, SDC: Screen-detected cancer, IC: Interval Cancer, TI: True interval, FN: False-negative, MS: Minimal-signs, OT: Occult tumors,

SFM: Screen-film mammography, DM: Digital mammography and HRT: Hormonal Replacement Therapy.
