# Supplementary material for: Impact of Risk Factors on Different Interval Cancer Subtypes in a Population-Based Breast Cancer Screening Programme
Source: PLoS One. 2014 Oct 21;9(10):e110207. doi: 10.1371/journal.pone.0110207 (PMC4204862; doi:10.1371/journal.pone.0110207)
Supplement: Table S2 — Crude hazard ratios from cause-specific survival analyses for incident screen-detected cancers and for interval cancer (overall and subtypes). (DOC) [file pone.0110207.s002.doc]

**Table S2.** Crude hazard ratios from cause-specific survival analyses for incident screen-detected cancers and for interval cancer (overall and subtypes).

|  | **Incident SDC** | **IC** | | **TI** | **FN** | | **MS** | **OT** | |
| --- | --- | --- | --- | --- | --- | --- | --- | --- | --- |
|  | HR* (95%CI) | HR* (95%CI) | | HR* (95%CI) | HR* (95%CI) | | HR* (95%CI) | HR* (95%CI) | |
| **Reading Method** |  |  | |  |  | |  |  | |
| Single vs Double | 0.93 (0.85-1.02) | 1.02 (0.89-1.16) | | 0.78 (0.60-1.02) | 0.85 (0.59-1.23) | | 0.38 (0.21-0.68) | 0.68 (0.38-1.21) | |
| **Type of mammogram** |  |  | |  |  | |  |  | |
| DM vs SFM | 1.53 (1.35-1.74) | 1.29 (1.06-1.57) | | 1.71 (1.25-2.35) | 1.61 (1.00-2.58) | | 0.65 (0.29-1.48) | 0.52 (0.16-1.63) | |
| **Early Recall** |  |  | |  |  | |  |  | |
| Yes vs No | 3.51 (3.10-3.98) | 1.10 (0.83-1.45) | | 1.10 (0.66-1.85) | 1.29 (0.64-2.62) | | 0.84 (0.31-2.28) | + | |
| **Previous false-positive** |  |  | |  |  | |  |  | |
| Yes vs No | 2.41 (2.13-2.72) | 2.63 (2.24-3.09) | | 2.27 (1.66-3.09) | 6.50 (4.73-8.93) | | 1.56 (0.84-2.89) | 0.34 (0.08-1.39) | |
| **HRT use** |  |  | |  |  | |  |  | |
| Yes vs No | 0.92 (0.81-1.04) | 1.18 (1.00-1.39) | | 1.23 (0.92-1.65) | 1.14 (0.73-1.78) | | 1.43 (0.91-2.22) | 0.84 (0.42-1.67) | |
| **Menopausal status** |  |  | |  |  | |  |  | |
| Premenopausal vs Postmenopausal | 0.86 (0.75-1.00) | 1.41 (1.20-1.65) | | 1.14 (0.84-1.54) | 1.25 (0.79-1.98) | | 0.77 (0.44-1.33) | 1.38 (0.82-2.33) | |
| **Family history of breast cancer** |  |  |  | | |  |  | |  |
| Yes vs No | 1.82 (1.64-2.02) | 1.67 (1.43-1.95) | | 1.98 (1.51-2.61) | 1.41 (0.90-2.22) | | 1.52 (0.93-2.48) | 1.41 (0.75-2.64) | |
| **Previous benign biopsy outside screening** |  |  |  | | |  |  | |  |
| Yes vs No | 1.38 (1.22-1.57) | 1.83 (1.55-2.15) | | 1.32 (0.96-1.83) | 2.21 (1.50-3.25) | | 1.49 (0.91-2.44) | 1.15 (0.55-2.38) | |

+ We excluded early recall, because there were no cases of early recall in occult tumors.

**Abbreviations**: SDC: Screen-detected cancer, IC: Interval Cancer, TI: True interval, FN: False-negative, MS: Minimal-signs, OT: Occult tumors, SFM: Screen-film mammography, DM: Digital mammography and HRT: Hormonal Replacement Therapy.
